# Asthma did not increase in‐hospital COVID‐19‐related mortality in a tertiary UK hospital

**DOI:** 10.1111/cea.13855

**Published:** 2021-03-02

**Authors:** Wei Chern Gavin Fong, Florina Borca, Hang Phan, Helen E. Moyses, Paddy Dennison, Ramesh J. Kurukulaaratchy, Hans Michael Haitchi

**Affiliations:** ^1^ School of Clinical and Experimental Sciences, Faculty of Medicine University of Southampton Southampton UK; ^2^ David Hide Asthma and Allergy Research Centre Isle of Wight NHS Trust Newport UK; ^3^ National Institute for Health Research (NIHR) Southampton Biomedical Research Centre (BRC) at University Hospital Southampton NHS Foundation Trust Southampton UK; ^4^ Clinical Informatics Research Unit, Faculty of Medicine University of Southampton Southampton UK; ^5^ Asthma, Allergy and Clinical Immunology Department University Hospital Southampton NHS Foundation Trust Southampton UK; ^6^ Institute for Life Sciences University of Southampton Southampton UK

To the Editor,

Asthma is the most prevalent chronic inflammatory respiratory disease worldwide affecting one in twelve adults (8.3%) in the United Kingdom (UK).^1^ Coronavirus disease 2019 (COVID‐19) has afflicted at least 80.3 million patients worldwide (17.3 million in the EU, 2.3 million in the UK) and has resulted in more than 1.6 million deaths (427,000 in the EU, 71,000 in the UK) (European Centre for Disease Prevention and Control: 30/12/2020). It is still unclear how asthma affects COVID‐19‐related mortality with marked regional differences noted.^2^ Comorbid associations of COVID‐19 with asthma were significantly lower than the prevalence of asthma in the regions studied.^3^ Additionally, COVID‐19 disease has not been shown to be more severe in patients with asthma^3^, ^4^ nor associated with increased mortality.^3^ To assess the relationship between asthma and in‐hospital COVID‐19‐related mortality, we conducted a retrospective analysis of the electronic healthcare record (EHR) at a large tertiary hospital in the South of England, University Hospital Southampton (UHS).

We retrospectively reviewed anonymized and non‐identifiable data from COVID‐19 Reverse Transcriptase‐Polymerase Chain Reaction (RT‐PCR) tested adult (age ≥18 years) patients with asthma (PWA) and patients with no asthma (PWNoA) who were admitted between 01 March and 31 May 2020. This was performed as part of an asthma service evaluation that was registered at UHS. Asthma status, demographics and co‐morbidities were based on ICD (International Classification of Disease) codes from the EHR. Asthma diagnosis reflected a physician diagnosis of asthma made in either Primary or Secondary Care following conventional diagnostic practice.^5^ Severe asthma (SA) was defined as patients managed with British Thoracic Society ‘high dose therapies’ and/or ‘continuous or frequent use of oral corticosteroids’.^5^ The primary outcome was all‐cause in‐hospital mortality during the most recent hospital stay. Competing‐risks survival regression was used to model time from admission to death. Discharge from hospital was specified as a competing risk, as discharged patients cannot experience in‐hospital death. Patients still in‐hospital on 31 May were censored. Clinical characteristics were compared using chi‐squared or Wilcoxon‐Mann‐Whitney test where appropriate. *p* values <.05 were regarded as significant. Statistical analysis was conducted using Stata (version 16, College Station, TX).

During our study period, there were 23,501 admissions to UHS, whereby 1916 (8%) were PWA, which was slightly below the adult asthma prevalence in the general UK population.^1^ A total of 6638 patients were tested for COVID‐19, whereby a larger proportion of PWA (914, 48%) were tested for COVID‐19 compared with PWNoA (5724, 27%). A significantly larger proportion of the PWA tested positive for COVID‐19 and a significantly greater proportion of them were admitted to the high dependency unit (HDU) (Table 1). Additionally, PWA were predominantly female, reflecting the higher prevalence of asthma among adult women. PWA were also more obese and less ethnically diverse. There were no differences between length or number of hospital stays, age or intensive care unit (ICU) admissions. Of the 914 PWA tested for COVID‐19, only 39 (4.3%) had SA, which mirrors the reported rate of 3%–10% in adults with asthma.^1^ Of the COVID‐19‐positive patients, 142 (27.6%) PWNoA, 32 (32.7%) mild/moderate PWA and 1 (25.0%) SA patient died in hospital.

**TABLE 1:** Clinical characteristics of COVID‐19 tested patients

	PWNoA	PWA	Overall	*p*‐Value
	*N* = 5724	*N* = 914	*N* = 6638	(PWNoA vs. PWA)
COVID−19 status, *n* (%) positive	515 (9.0%)	102 (11.2%)	617 (9.3%)	**.04**
Age, median (IQR)	65 (42, 79)	64 (47, 78)	65 (42, 79)	.6
Gender, *n* (%) Male	2707 (47.3%)	372 (40.7%)	3079 (46.4%)	**<.001**
Obese, *n* (%)	137 (2.4%)	341 (37.3%)	478 (7.2%)	**<.001**
BAME, *n* (%)^a^	373 (6.5%)	38 (4.2%)	411 (6.2%)	**.002**
No. of hospital stays, median (IQR)	1 (1, 2)	1 (1, 2)	1 (1, 2)	.2
Single hospital stay, *n* (%)	4037 (70.5%)	631 (69.0%)	4668 (70.3%)	.4
Length of stay (days), median (IQR)	2 (1, 7)	2 (0, 7)	2 (1, 7)	.2
Admitted to HDU only, *n* (%)	195 (3.4%)	44 (4.8%)	239 (3.6%)	**.03**
Admitted to ICU only, *n* (%)	495 (8.7%)	78 (8.5%)	573 (8.6%)	.9
Admitted to HDU AND ICU, *n* (%)	93 (1.6%)	23 (2.5%)	116 (1.8%)	.06

Note

Data were analysed using chi‐squared or Wilcoxon‐Mann‐Whitney test where appropriate. *p* Values <.05 were regarded as significant.

Abbreviations: BAME, Black Asian and minority ethnicities; COVID‐19, coronavirus disease 2019; HDU, High dependency unit; ICU, intensive care unit; IQR, interquartile range; *N and n*, number; PWA, patients with asthma; PWNoA, patients with no asthma.

The bold values mean that the comparisons are statistically significant of less than .05.

To adjust for clinically relevant variables for COVID‐19‐related death such as age, gender, ethnicity, obesity, SA and other co‐morbidities (Table 2), an adjusted competing‐risks regression analysis was performed. In this model, COVID‐19 positivity, independent of any co‐morbidities, increased the rate of in‐hospital mortality fourfold (Subdistribution Hazard Ratio [SHR]: 4.50; 95 CI = 3.49–5.80, *p* < .001). Asthma and SA were not associated with increased rate of in‐hospital death while age, male gender, HDU and ICU admissions were (Table 2).

**TABLE 2:** Results of competing‐risks regression for mortality

	SHR (95% CI)	*p*‐Value
Unadjusted (*N* = 6638)		
Asthma status (asthma vs. no asthma)	1.04 (0.81, 1.34)	.75
COVID−19 (positive vs. negative)	6.19 (5.14, 7.45)	**<.001**
Fully adjusted (*N* = 6008)		
Asthma status (asthma vs. no asthma)	1.07 (0.81, 1.41)	.65
COVID−19 (positive vs. negative)	4.50 (3.49, 5.80)	**<.001**
Age	1.05 (1.05, 1.06)	**<.001**
Gender (male vs. female)	1.41 (1.16, 1.73)	**.001**
BAME (yes vs. no)	0.82 (0.51, 1.30)	.39
Obesity (yes vs. no)	0.99 (0.71, 1.38)	.96
No. of hospital stays	1.02 (0.96, 1.08)	.56
Severe asthma (yes vs. no)	0.87 (0.16, 4.67)	.87
HDU (yes vs. no)	2.62 (1.91, 3.58)	**<.001**
ICU (yes vs. no)	1.81 (1.33, 2.45)	**<.001**
HDU & ICU interaction	0.25 (0.11, 0.58)	.001
Hypertension, (yes vs. no)	0.88 (0.66, 1.18)	.40
Hyperlipidaemia, (yes vs. no)	0.71 (0.50, 1.02)	.06
Diabetes, (yes vs. no)	1.05 (0.77, 1.43)	.77
Smoking, (yes vs. no)	0.95 (0.62, 1.45)	.81
Alcohol dependence, misuse or alcohol related disease, (yes vs. no)	0.71 (0.44, 1.12)	.14
Ischaemic heart disease, (yes vs. no)	0.97 (0.73, 1.29)	.82
Heart failure, (yes vs. no)	1.33 (0.99, 1.80)	.06
Peripheral vascular disease, (yes vs. no)	1.08 (0.80, 1.46)	.63
Cerebrovascular disease, (yes vs. no)	1.11 (0.83, 1.49)	.48
Renal disease, (yes vs. no)	1.00 (0.75, 1.35)	.98
COPD, (yes vs. no)	1.07 (0.76, 1.51)	.69
Liver disease, (yes vs. no)	1.24 (0.75, 2.08)	.40
Allergic rhinitis, (yes vs. no)	0.97 (0.53, 1.77)	.91
Neoplasms, (yes vs. no)	0.98 (0.75, 1.27)	.87
Dermatitis and eczema, (yes vs. no)	1.23 (0.81, 1.89)	.33
Allergy, (yes vs. no)	1.32 (0.87, 2.01)	.20

Abbreviations: BAME, Black Asian and minority ethnicities; COPD, Chronic obstructive pulmonary disease; COVID‐19, coronavirus disease 2019; HDU, High dependency unit; ICU, intensive care unit; SHR, Subdistribution Hazard Ratio; N, number.

Hazard ratios for co‐morbidities are comparing co‐morbidity present vs. not present.

The bold values mean that the comparisons are statistically significant of less than .05.

Our findings suggest that PWA were more likely to test positive for COVID‐19. However, this most likely reflects surveillance bias as a larger proportion of PWAs were tested for COVID‐19, compared with PWNoA. Alternatively, it may be explained by PWA having deficiencies in their innate immunity^6^ and therefore greater susceptibility to respiratory viral infections. Nonetheless, holding all other variables constant, in‐hospital mortality of PWAs was comparable to PWNoA, which is consistent with wider national primary care findings.^7^ However, COVID‐19 infection, age, male gender and HDU/ICU admission conferred significantly increased risk for in‐hospital death. This strongly supports the concept that adhering to measures that limit spread of COVID‐19 could save lives and reduce the burden on the National Health Service, independent of asthma status.

Severe asthma was not associated with increased risk for in‐hospital death in our adjusted model. However, this may be due to small numbers as of the 39 tested SA patients, four tested positive for COVID‐19 and one of whom died. Alternatively, it could be part‐explained by the fact that our SA clinics at UHS were switched to remote consultations and offered intensified virtual patient access during the height of the pandemic. Furthermore, 55% of our SA patients formally shielded according to government mandate Additionally, our SA patients were on high dose inhaled corticosteroids, namely ciclesonide which has been shown to inhibit SARS‐CoV‐2 replication *in vitro* and attracted interest as a potential COVID‐19 therapy.^8^ Our findings for SA mortality were consistent with recently published findings from the UK Severe Asthma Registry.^9^

Similar to other studies,^3^, ^4^, ^7^ we showed that age and male gender were associated with an increased mortality rate. However, unlike other studies, ethnicity, obesity, hypertension and cardiovascular disease were not. These differences may reflect limitations inherent to retrospective EHR analyses with missing data and hospital coding inaccuracies or the characteristics of the local population served by UHS. Additionally, while our findings are representative of our tertiary centre during the ‘first COVID‐19 wave’, it may not be translatable to different populations and centres. A similar national, international and multi‐centre analysis is needed to clarify the relationship between asthma and COVID‐19 at a broader population level.

In conclusion, this retrospective review during the first wave of the pandemic confirmed that COVID‐19 infection independently associated with increased in‐hospital mortality. Additionally, we found that during the first wave of the pandemic, while more admitted PWA tested COVID‐19 positive, which was likely due to surveillance bias, they did not have increased in‐hospital mortality compared with PWNoA. Furthermore, SA status did not increase in‐hospital mortality. These findings may have been influenced by a variety of mitigating factors and merit further assessment in any subsequent waves of the COVID‐19 pandemic.

## CONFLICT OF INTEREST

The authors declare no conflict of interest.

## AUTHOR CONTRIBUTIONS

WCGF contributed to conception and design, analysis and interpretation of data and co‐wrote the manuscript. FB and HP contributed to acquisition of data from the electronic health records and interpretation of data. HEM contributed to statistical analysis and interpretation of the data. PD and RJK contributed to the interpretation of data. HMH developed the concept and design, contributed to analysis and interpretation of the data and co‐wrote the manuscript. All authors provided critical revision of the manuscript for important intellectual content.

## Data Availability

The data that support the findings of this study are available on request from the corresponding author. The data are not publicly available due to privacy or ethical restrictions.
